# Cross-organelle communication at the core of longevity

**DOI:** 10.18632/aging.101373

**Published:** 2018-01-22

**Authors:** Nuno Raimundo, Anita Kriško

**Affiliations:** 1 Institut für Zellbiochemie, Universitätsmedizin Göttingen, Göttingen, Germany; 2 Mediterranean Institute for Life Sciences, Split, Croatia

**Keywords:** aging, proteostasis, mitochondria, yeast, *C. elegans*, crosstalk

Complex signaling networks are needed for coordination of biological processes, such as development, physiological homeostasis, as well as aging. Selection for survival has led to the evolution of cellular maintenance machineries to coordinate and integrate biological processes and regulate cell fate. While age-dependent changes have usually been studied individually, recent developments call for an integrated exploration of the communication between key pathways relevant for aging.

Several cellular organelles and processes are involved in stress responses and aging, implying coordination between different subcellular structures and signaling networks. Decades of research have identified nine hallmarks of aging, common across species: genomic instability, telomere attrition, epigenetic alterations, loss of proteostasis, deregulated nutrient sensing, mitochondrial dysfunction, cellular senescence, stem cell exhaustion, and altered intercellular communication [1]. The importance of protein maintenance in normal cellular functioning, as well as during aging, is indisputable. However, the relationships between proteostasis and other aging hallmarks remain largely unaddressed.

In particular, little is known about the communication of the protein chaperone network with the cellular metabolic activity and mitochondrial function. Using budding yeast, *Saccharomyces cerevisiae*, we have gathered multiple lines of evidence demonstrating that the mitochondrial and metabolic activity can be modulated by the status of protein folding environment [2, 3]. We, therefore, posit that the changes in the proteostasis status and metabolism are concerted with a common task of safeguarding cellular fitness and longevity. The metabolic response to improved protein maintenance (via enrichment in chaperone expression) phenocopies the glucose starvation response [3]. Target of Rapamycin (TOR) pathway emerges as a hub of this signaling network, itself being responsive of the protein folding environment (via Hsp82 expression level) and responsible for relaying the message to glucose metabolism and mitochondrial activity by acting as a negative regulator of Snf1/AMPK (5’ AMP-activated protein kinase). Importantly, the replicative lifespan extension in strains with chaperone enrichment is abrogated when the proteostasis-metabolism crosstalk is uncoupled. This suggests that the ensemble of metabolic changes triggered by TORC1 deactivation is essential for chaperone-mediated RLS extension, implying that improved proteostasis is not acting independently to promote longevity.

Cell-wide metabolic reprogramming also plays a role in mitigating compartmentalized proteotoxicity [2]. Regardless of the compartment of proteotoxic stress origin (mitochondria, endoplasmic reticulum, cytosol), cells activate a common cross-organelle response (CORE). In addition to protein maintenance activation in the cytosol, mitochondria and the ER, CORE features metabolic reprogramming and repression of mitochondrial respiration with a likely role of contributing to management of proteotoxicity and to cellular resilience during stress.

Several recent reports go along similar lines of demonstrating the communication between compartmentalized proteostasis. Kim et al. have reported the existence of a novel pathway, mitochondrial-to-cytosolic stress response (MCSR), required in linking the mitochondrial and cytosolic protein folding environments in *Caenorhabditis elegans* [4]. Notably, mitochondrial proteotoxic stress triggers an increase in fatty acid synthesis leading to atypical lipid accumulation, which has been proven indispensable in triggering the MCSR. Both *C. elegans*, as well as human cells benefit from the MCSR induction: the stress response is protective against the proteotoxicity originating from polyglutamine aggregation. Moreover, mitochondrial stress response, comprising mitochondrial proteostasis, is activated in response to cytosolic amyloid β aggregation (Aβ), across species [5]. Aβ accumulation induces UPR^mt^ as well as mitophagy, and the crosstalk is likely enabled via alterations in the function of mitochondrial import machinery. The opposite also stands: improving mitochondrial proteostasis leads to a decline in cytosolic Aβ aggregation and favors worm survival and lifespan [5]. These effects closely match the described CORE pathway [2].

A decline in the activity of the heat shock response (HSR) is typically observed during aging in adult *C. elegans*. In a recent report, Labbadia et al. have found that the electron transport chain regulates the HSR activity [6]: mild mitochondrial stress maintains the HSR active even in adulthood, leading to the prevention of proteostasis collapse during aging (observed as a decline in polyglutamine aggregation) and preservation of healthspan in *C. elegans*. In a similar fashion, short-term mitochondrial stress in mammalian cells has been shown to trigger lysosomal biogenesis and stimulate autophagy [7]. Given the role of autophagy in the clearance of protein aggregates, this mitochondria-lysosome-autophagy-proteostasis axis illustrates the role of cross-organelle communication in cell stress response.

The crosstalk of mitochondria with other organelles as well as with protein maintenance machineries is proving to be crucial for cellular robustness and survival. However, it only started being explored, and the full outreach of this crosstalk is far from understood. Its exact biological role remains to be investigated in further detail, likely providing novel insights into basic cellular functioning, and shedding new light on the process of aging, as well as neurodegenerative diseases. Although the traditional strategy of narrow focus and single-level approaches has unquestionably led to significant progress and remarkable insights, it is nevertheless becoming clearer that a more integrated approach is needed if we are to obtain fundamental understanding of aging and healthspan.
